# Preface to “A Commemorative Issue in Honour of Professor Nick Hadjiliadis: Metal Complex Interactions with Nucleic Acids and/or DNA”

**DOI:** 10.3390/ijms19123815

**Published:** 2018-11-30

**Authors:** Christina N. Banti, Sotiris K. Hadjikakou

**Affiliations:** Section of Inorganic and Analytical Chemistry, Department of Chemistry, University of Ioannina, 45110 Ioannina, Greece; cbanti@cc.uoi.gr

This Special Issue of the *International Journal of Molecular Science* comprises a comprehensive study on "Metal Complex Interactions with Nucleic Acids and/or DNA". This Special Issue has been inspired by the important contribution of Prof. Nick Hadjiliadis in the field of palladium or/and platinum/nucleic acid interactions. It covers a selection of recent research and review articles in the field of metal complex interactions with nucleic acids and/or DNA.

Metal complexes have long been recognized as critically important components of nucleic acid chemistry, both in the regulation of gene expression and as promising therapeutic agents. The ability to recognize and understand how metal complexes interact with DNA at the molecular level has become an active research area at the interface between biological inorganic chemistry, molecular biology, and medicine. Arguably the most prominent drug which contains a metal is cisplatin, the most widely used anti-cancer drug. The success of cisplatin in chemotherapy and the clarification of its mechanism of action through its interaction with DNA has motivated a large number of studies on metal complex interactions with nucleic acids or/and DNA. Thus, the reader of this Special Issue will gain an appreciation of the real role of the interactions of metal complexes with nucleic acids or/and DNA in modern medicine.

This Special Issue on "Metal complex Interactions with Nucleic Acids and/or DNA" provides an overview of this increasingly diverse field, presenting recent developments and the latest research with particular emphasis on metal-based drugs and metal ion toxicity.

Inorganic biochemistry or bioinorganic chemistry is a multidiscipline field which involves inorganic chemistry, biochemistry, spectroscopy, material science, biology, and medicine. The introduction of metal ions or metal ion binding components into a biological system for the treatment of diseases is one of the main subdivisions in the field of inorganic biochemistry. Nowadays, at the forefront of the field is the development of new metallodrugs for diagnostics (radiopharmaceuticals drugs), medicines (anticancer, antimicrobial/antiparasitic, therapeutic radiopharmaceuticals, photochemotherapeutic metallodrugs, antiarthritic, antidiabetes, antiviral, metallodrugs addressing deficiencies syndromes), and tools for chemical biology, biocatalysis, and bioelectronics, as well as the characterization of metalloproteins, enzymes, and their model complexes. Recently, biomaterials have been applied in many cases such as cardiovascular medical devices, orthopedic and dental applications, ophthalmologic applications, bioelectrodes and biosensors, burn dressings and skin substitutes, sutures, and drug delivery systems. For the coming years, the well-defined bioavailability, absorption, distribution, metabolism, and excretion of metal-based drugs will be the main target of new metallodrugs. The use of nanoparticles, micelle emulsions, and liposomal formulations can open new opportunities for improved delivery, cell uptake, and targeting. 

Emeritus Professor Nick Hadjiliadis is one of the pioneers in the field of bioinorganic chemistry. He graduated from the University of Athens, and subsequently earned a Masters in Science from the University of Montreal and a Doctor of Chemistry, PhD from the University of Montreal in 1975. He served as a Professor of Inorganic and General Chemistry at the Department of Chemistry of the University of Ioannina, Greece, since 1980. He worked on the interaction of metal ions with nucleobases and nucleosides, as well as with DNA and RNA. This pioneering work led to the first conclusions elucidating the mechanism of the antitumoral action of cisplatin. His research interests also included: (i) the synthesis, characterization, and study of the antitumor properties of new metal complexes (e.g., Pt, Pd, Sn, Sb, etc.); (ii) the study of metalloenzyme models and the clarification of their mechanism of action. His research on thiamine enzymes in the presence of divalent metal ions, for which he proposed a mechanism, was also a pioneering work in the field of enzyme/metal ion interactions. He also studied (iii) the mechanism of action of superoxide dismutase, Cu(II), and Zn(II); (iv) peptide interactions with metal ions, as models of enzymes or other biological systems; (v) the involvement of metal ions such as Ni(II) and Cu(II) in carcinogenesis; (vi) the biocatalysis of new materials; (vii) the mechanism of action of anti-thyroid drugs; (viii) organometallic chemistry, etc. He has numerous publications (up to 260), which have been cited more than 7000 times with an h-index of 44. He has been invited to 70 international conferences (as a Lead Speaker). He has coordinated many research and teaching programs. Important international conferences dedicated to the field of inorganic biochemistry—such as 5-ISABC, HALCHEM-III, 12-EURASIA, etc.—have been organized and chaired by him. Furthermore, he mentored many scientists, initiating their careers as academicians worldwide. Special attention should be paid to his contribution to the foundation and operation of the Interdisciplinary Program of Postgraduate Studies in Bioinorganic Chemistry, which he led for a decade. His contribution to the field of Inorganic-Bioinorganic Chemistry is undoubtedly superior in quality and unique for a Greek scientist. Overall, he contributed to the progress of inorganic chemistry not only at the University of Ioannina, Greece, but in the whole nation.

Recognizing this contribution of Emeritus Professor Nick Hadjiliadis to the field of inorganic biochemistry and especially to the field of palladium or/and platinum/nucleic acid interactions, it is our honour to dedicate the prologue of this commemorative issue of the *International Journal of Molecular Sciences* to him.

This Special Issue is composed of 14 articles, which are briefly reviewed below.

Yu-Wen Chen et al. showed that the i-motif DNA sequence may transition to a base-extruded duplex structure with a GGCC tetranucleotide tract when it is bound to the (Co^II^)-mediated dimer of chromomycin A3 [1]. G. Momekov et al. investigated two paramagnetic palladium(III) complexes of hematoporphyrin IX for their ability to process DNA adducts as well as for their antineoplastic and apoptogenic activities [2]. E. Makkonen et al. reported a combined quantum mechanics/molecular mechanics molecular dynamics and time-dependent density functional study of silver-mediated deoxyribonucleic acid nanostructures [3]. S. Liu et al. investigated the interactions between ruthenium(II) complexes and 15-mer single- and double-stranded oligodeoxynucleotides and they tested the thermodynamic base and sequence selectivity [4]. G.K. Latsis et al. investigated two polyorganotic acetate complexes against DNA with possible implementation towards breast cancer cells [5]. S. Savino et al. tested the ability of platinum prodrugs of kiteplatin with a-lipoic acid in the axial position to target in mitochondria [6]. Q.Y. Yang et al. tested the molecular mechanism of two transition metal complexes with 2-((2-(pyridin-2-yl)hydrazono)methyl)quinolin-8-ol against tumor cells [7]. M. Hande et al. described the synthesis and hybridization properties of short oligonucleotides incorporating cyclopalladated benzylamine “warheads” at their 5′-termini [8]. M.F. AlAjmi et al. evaluated the benzimidazole-derived biocompatible copper(II) and zinc(II) complexes as anticancer chemotherapeutics [9]. T. Qin et al. examined the binding of zinc cationic porphyrin towards B-DNA and Z-DNA [10] A.B. Olejniczak et al. presented an overview of the methods for incorporating metal centers into nucleic acids based on metal–boron cluster complexes (metallacarboranes) as the metal carriers [11]. Y.H. Lai et al. reviewed the mechanisms by which the regulation of copper homeostasis modulates the chemosensitivity of tumors to platinum drugs [12]. V. Murray et al. reviewed the abilities of bleomycin to interact with DNA [13]. N.C. Sabharwal et al. investigated the interactions between spermine-derivitized tentacle 2 porphyrins and the human telomeric DNA G quadruplex [14].
